# Supplementary figures and images for: Effectiveness of Preoperative Immunonutrition in Improving Surgical Outcomes after Radical Cystectomy for Bladder Cancer: Study Protocol for a Multicentre, Open-Label, Randomised Trial (INu-RC)
Source: Healthcare (Basel). 2024 Mar 20;12(6):696. doi: 10.3390/healthcare12060696 (PMC10970425; doi:10.3390/healthcare12060696)

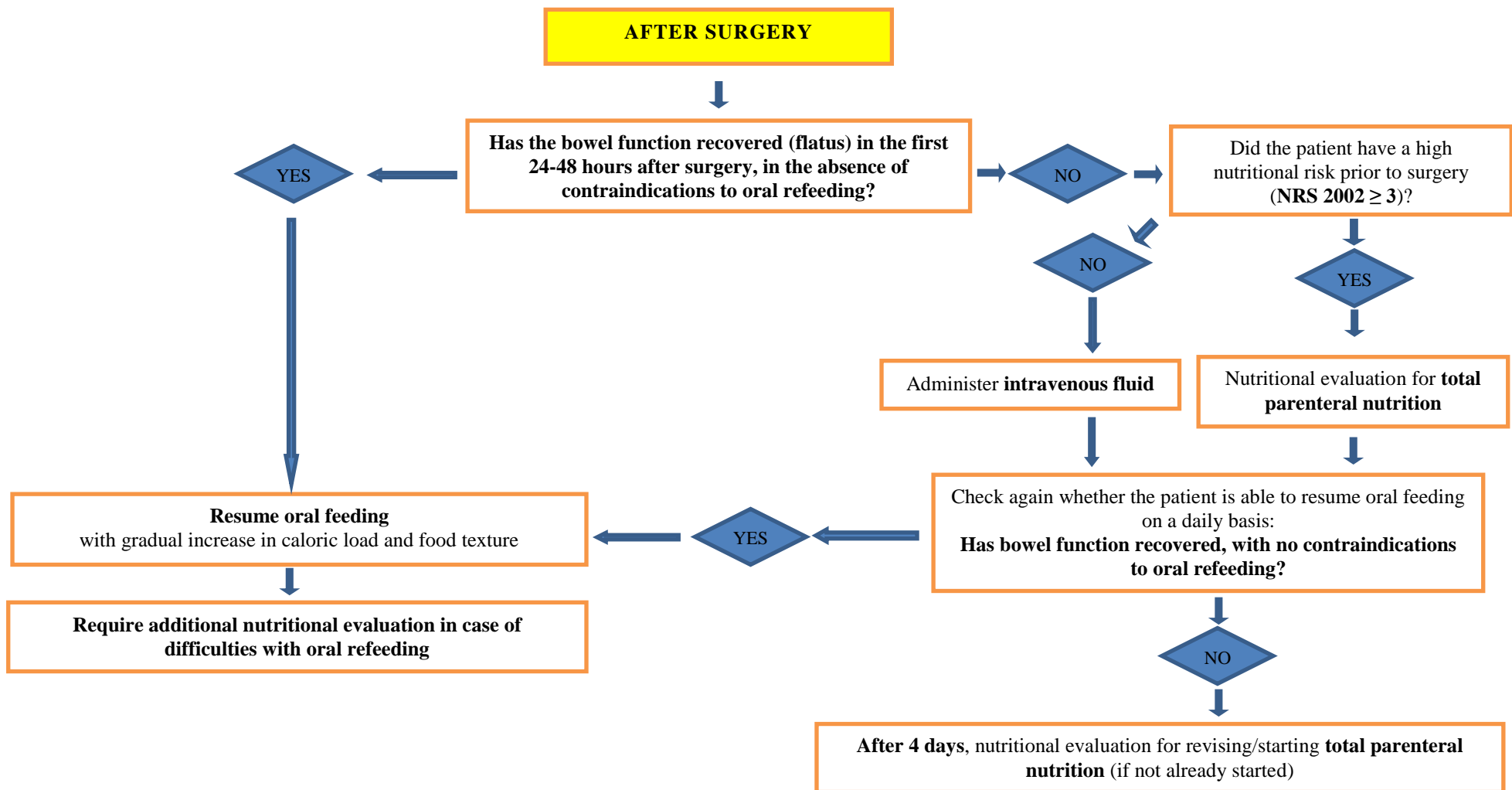

Supplement: Supplementary file 1 [file healthcare-12-00696-s001.zip › healthcare-2828848-supplementary.pdf]
